# Balanced Convolutional Neural Networks for Pneumoconiosis Detection

**DOI:** 10.3390/ijerph18179091

**Published:** 2021-08-28

**Authors:** Chaofan Hao, Nan Jin, Cuijuan Qiu, Kun Ba, Xiaoxi Wang, Huadong Zhang, Qi Zhao, Biqing Huang

**Affiliations:** 1 Department of Automation, Tsinghua University, Beijing 100084, China; hcf20@mails.tsinghua.edu.cn (C.H.); bk19@mails.tsinghua.edu.cn (K.B.); 2 Chongqing Center for Disease Control and Prevention, Department of Occupational Health and Radiation Health, Chongqing 400042, China; jinnan@chongqingcdc.cn (N.J.); qiucuijuan@chongqingcdc.cn (C.Q.); wangxiaoxi@chongqingcdc.cn (X.W.); zhanghuadong@chongqingcdc.cn (H.Z.)

**Keywords:** convolutional neural networks, pneumoconiosis detection, interpretability, balanced training

## Abstract

Pneumoconiosis remains one of the most common and harmful occupational diseases in China, leading to huge economic losses to society with its high prevalence and costly treatment. Diagnosis of pneumoconiosis still strongly depends on the experience of radiologists, which affects rapid detection on large populations. Recent research focuses on computer-aided detection based on machine learning. These have achieved high accuracy, among which artificial neural network (ANN) shows excellent performance. However, due to imbalanced samples and lack of interpretability, wide utilization in clinical practice meets difficulty. To address these problems, we first establish a pneumoconiosis radiograph dataset, including both positive and negative samples. Second, deep convolutional diagnosis approaches are compared in pneumoconiosis detection, and a balanced training is adopted to promote recall. Comprehensive experiments conducted on this dataset demonstrate high accuracy (88.6%). Third, we explain diagnosis results by visualizing suspected opacities on pneumoconiosis radiographs, which could provide solid diagnostic reference for surgeons.

## 1. Introduction

### 1.1. Pneumoconiosis Diagnosis

Pneumoconiosis is a disease caused by long-term inhalation of mineral dust [1]. Its retention in the lungs during occupational activities, mainly characterized as diffuse fibrosis of lung tissue, is the most serious and common occupational disease in China. The high prevalence and costly treatment of pneumoconiosis bring huge economic losses to society. According to the national occupational disease report, by the end of 2018, more than 970,000 cases of occupational diseases were reported in China, and more than 870,000 cases of pneumoconiosis were included, accounting for about 90% of all occupational disease cases. Since 2010, the number of new pneumoconiosis cases reported each year has exceeded 20,000 cases. According to relevant surveys, the average annual medical cost per pneumoconiosis case in China is 19.05 thousand yuan, and other indirect costs are 45.79 thousand yuan on average. Simplifying an average survival period after diagnosis as 32 years, the average economic burden caused by pneumoconiosis for each patient is 2.075 million yuan without taking inflation into account [2,3].

Though pneumoconiosis is prevalent and costly, many cases have confirmed that the earlier that pneumoconiosis is diagnosed and treated, the better treatment could be. The main cause of death in cases of pneumoconiosis lies in a variety of complications that emerge in the late-developed stage, of which respiratory complications account for 51.8% and cardiovascular disease complications account for 19.9%. Early diagnosis and treatment of pneumoconiosis will largely inhibit the development of complications, which is of great importance for treatment.

In China, the diagnosis of pneumoconiosis based on chest X-ray radiographs is still manual in clinical practice, rather than computer-aided and automatic diagnosis, which creates two drawbacks. First, the accuracy rate is not high enough. Manual radiograph reading requires high diagnostic skills, and the variation in diagnosis of pneumoconiosis caused by inconsistency of professional level and experience can be as high as 75.6%. Second, stability is not good enough. When workload is high, physicians may overlook subtle lesions due to fatigue, some of these being small pulmonary nodules and subtle calcified spots. Therefore, in order to improve the accuracy and stability of pneumoconiosis diagnosis, there are two major bottlenecks that need to be addressed, and an automatic and data-driven pneumoconiosis diagnosis system will make early and accurate diagnosis possible.

### 1.2. Data-Driven Methods and Deep Learning

In the literature on the computer analysis scheme of chest radiographs in the twentieth century, three main areas are distinguished by Ginneken et al. [4]: (1) general processing techniques, (2) algorithms for segmentation and (3) analysis for a particular application. However, these methods emphasize utilization of imaging processing techniques, rather than data mining and pattern recognition. Electronic health records provide massive image data and rich patient information, especially chest radiographs and graphic details, which make data-driven methods possible.

Recent research has shown that a data-driven automatic diagnostic system can be simplified to a framework in which image features and texture patterns of chest radiographs are first extracted, followed by a data-driven classifier based on a machine learning algorithm. Yu et al. first enhanced opacity details on images by applying a multi-scale difference filter bank algorithm, and then they calculated histogram features and co-occurrence matrices as artificially encoded information [5]. Zhu et al. utilized 22 wavelet-based energy texture features. Then, they applied a support vector machine (SVM) to distinguish between normal and abnormal samples and reached an AUC of 0.974 ± 0.018 and accuracy of 0.929 ± 0.018 [6]. In addition, Zhu et al. compared the classification ability of decision tree (DT) and support vector machine (SVM) with four different kernels for pneumoconiosis diagnosis, and they finally reached the conclusion that the AUCs of DT and SVM were 0.88 and 0.95, respectively. Furthermore, among all tested SVM kernels, polynomial kernel has performed best [6]. Researchers [7] also have utilized three-stage artificial neural network (ANN) for hierarchical classification, while four extracted features are still calculated in fixed paradigm, including gray-level histogram, gray-level difference histogram, gray-level co-occurrence matrix (GLCOM) feature map and gray-level run-length matrix (GLRLM) feature map in each ROI, which is still not end-to-end.

In 1998, inspired by individual neurons in the primary visual cortex of cats, Yann LeCun et al. proposed LeNet [8], the first modern convolutional neural network, to classify handwriting digits, which provides an end-to-end differentiable model for image classification. Convolutional neural networks (CNNs) differ from other neural network models that have convolutional operations as the main character. In 2012, AlexNet [9], the latest CNN at that time, outperformed the second-place system by 12% in the ImageNet image classification competition. Since then, CNNs have been widely studied and largely improved. By now, researchers have proposed ZFNet [10], VGGNet [11], GoogleNet [12], ResNet [13], DenseNet [14], EfficentNet [15] and many other deep convolutional structures, which are called deep learning models. CNNs provide end-to-end solutions for image feature extraction and outperform traditional benchmarks in nearly all image recognition tasks, for instance, image classification, semantic segmentation, image retrieval and object detection.

Recently, data-driven deep learning has made achievements in assisting physicians with lung disease diagnosis. Cai et al. applied texture analysis in pneumonia diagnosis [16] and achieved an accuracy of 0.793 on diagnosing 29 images. Kermany et al. established a deep learning framework for pneumonia diagnosis and applied it to CT and X-ray datasets [17]. They demonstrated that performance of diagnosis based on deep learning is comparable to that of human experts. Specifically in silicosis diagnosis, Wang XH et al. investigated the powerful capability of deep learning and demonstrated that the performance of Inception-V3 is better than that of two certified radiologists [18]. However, detection performance and balance between accuracy and recall still have much room for improvement. Moreover, lack of interpretation impedes CNNs from playing a part in clinical application.

In summary, contributions of this paper include the following:

(1) We have proposed a pneumoconiosis radiograph dataset based on electronic health records provided by Chongqing CDC, China, which is a full image dataset under privacy protection guidelines. The URL is https://cloud.tsinghua.edu.cn/f/d8324c25dbb744b183df/ (accessed on 14 August 2021)
(2) We have established two data-driven deep learning models based on ResNet and DenseNet, respectively. A brief comparison and discussion has been conducted on their performance. We rebalance weights of positive and negative samples, which trade off well between accuracy and recall.
(3) We have explained diagnosis results by interpreting feature maps and visualizing suspected opacities on pneumoconiosis radiographs, which could provide a solid diagnostic reference for surgeons.

## 2. Materials and Methods

### 2.1. Dataset Preparing Process

Chongqing CDC, China, has collected chest radiograph files for approximately one year from August 2016 to June 2017, which are important components of electronic health records. For privacy protection, we removed patient names and other private information from these files, and we then invited two clinical experts to diagnose pneumoconiosis cases as data annotation. We converted the above medical image files from DICOM format to jpg format, and in this way images could be shown and processed by the algorithm more easily [19]. We eliminated low-quality or irrelevant images of the same patient and removed images with omissive or incorrect annotation from the dataset. An original radiograph dataset with 706 images was finally obtained, of which 142 cases were pneumoconiosis-positive and the rest were negative.

We then conducted some pre-processing and refining to the above images.

**Figure 1:**
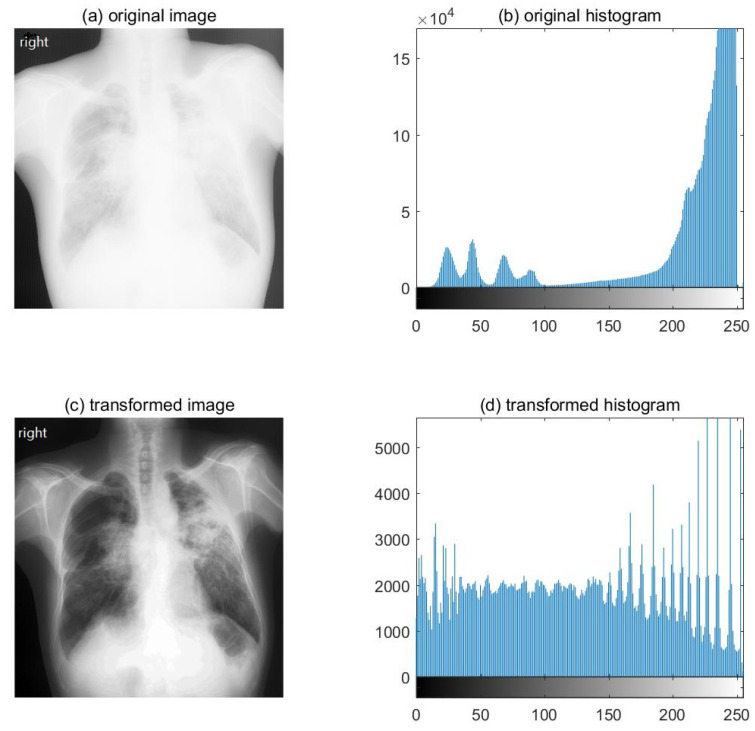
Image and histogram before and after histogram equalization. (**a**) Original image; (**b**) original histogram; (**c**) transformed image and (**d**) transformed histogram after histogram equalization.

First, we enhanced image contrast by using histogram equalization to highlight possible features. Histogram equalization is a technique used to adjust pixel distribution and allocate image intensities [20]. Figure 1 shows the image and histogram before and after histogram equalization, which demonstrates that the contrast of the new image has been enhanced and its histogram has also been equalized.

Second, we downsized images to one quarter of their original size, and we finally obtained images that were 694 × 719 pixels. This radiograph shrink operation reduces storage space and increases program speed, while preserving details to the maximum extent.

Finally, all images were randomly split into a training set and testing set based on a four-to-one ratio. The training set consisted of 452 negative samples and 114 positive samples, while the testing set consisted of 112 negative samples and 28 positive samples. We published data at https://cloud.tsinghua.edu.cn/f/d8324c25dbb744b183df/ (accessed on 14 August 2021).

This study was approved by the Medical Ethics Committee of Chongqing Center for Disease Control and Prevention (17 May 2019) and the Academic Management Committee of Chongqing Center for Disease Control and Prevention (17 May 2019). Informed consent was obtained from all physician participants involved in the study. Patient consent was waived due to the retrospective nature for use of their data.

**Figure 2:**
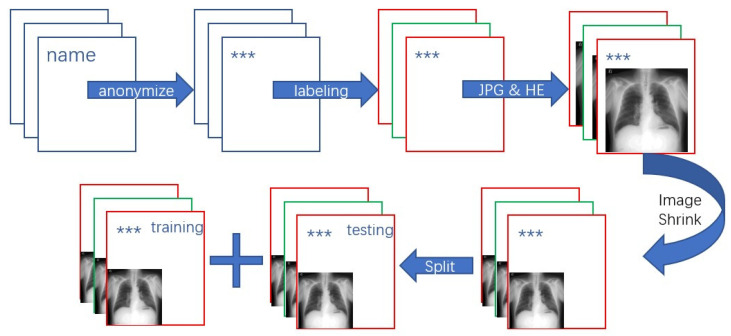
Preparation procedure of the pneumoconiosis radiograph dataset.(“***” represents for anonymization).

Figure 2 shows the preparation procedure of our pneumoconiosis radiograph dataset.

### 2.2. Convolutional Models: ResNet and DenseNet

Convolutional neural networks (CNNs) have excellent performance in many fields, especially in image-related tasks such as image classification [9,21], object detection [22] and semantic segmentation [23]. CNNs are mainly composed of three types of neural network layers, namely the convolutional layer, pooling layer and full connection layer.

Convolutional layers adaptively extract features from input data through convolution operation with kernels of different sizes. Let input data for a convolutional layer be a tensor with four dimensions, $\left(N,C_{in},H_{in},W_{in}\right)$ and output data be a tensor $\left(N,C_{in},H_{in},W_{in}\right)$, where *N* represents batch size, *C* number of channels, *H* height of input data and *W* width of input data. The calculation method of each dimension of output data is shown as Equations (1) and (2):

(1) $$out\left(N,C_{out}\right)=bias\left(C_{out}\right)+\Sigma_{0}^{C_{in}}weight\left(C_{out},k\right)*input,$$

(2) $$H_{out}\left(W_{out}\right)=\frac{H_{in}\left(W_{in}\right)+2\times padding-kernelsize}{stride}+1,$$

where *weight* represents the convolutional parameter, *padding* means width of zero padding that ensures smooth convolution at the edge, *kernel size* is the size of the convolutional kernel and *stride* is the moving step size of the convolutional kernel. Between convolutional layers, a nonlinear activation function is implemented to improve fitting ability, for instance, sigmoid function [24,25], tanh function and Rectified Linear Unit function (ReLU) [26].

Pooling layers include max pooling and average pooling [27], picking the maximum and calculating the average of the selected pooling region, respectively. Pooling operation has advantages that make it almost essential in CNNs. It helps in reducing the size of feature maps, narrowing the quantity of network parameters, improving computing speed and inhibiting overfitting.

Fully connected layers function as a classification or regression head. Going through convolutional and pooling layers, the final feature map is flattened into a high dimension vector, and then it is remapped by fully connected layers to one hot-label space or metric space to complete classification or regression, respectively.

Since LeNet [8] and AlexNet [9], CNNs have been widely studied and largely improved. Fast operation, light deployment and high precision are key objectives when designing CNNs, and two variants of CNNs, ResNet [13] and DenseNet [14], have received much attention. We implemented these two convolutional models in pneumoconiosis detection and obtained high accuracy after refining and rebalancing.

#### 2.2.1. ResNet

Let $x_{l-1}$ and $x_{l}$ represent feature maps after the (*l* − 1)th and *l*th convolutional layers, and $H_{l}$ the *l*th convolutional layer. The traditional CNNs follow the equation below (3):

(3) $$x_{l}=H_{l}\left(x_{l-1}\right).$$

To obtain high precision, the number of convolutional layers increases and CNNs go deep to extract features. However, because of the exponential effect of the chain derivative rule in the back propagation algorithm, network degradation happens. The gradient attenuates exponentially, and weights and parameters cannot be updated, leading to failure of optimization. When network degradation happens, both training loss and test loss increase. It differs from overfitting [28], in which case training loss decreases while test loss increases.

**Figure 3:**
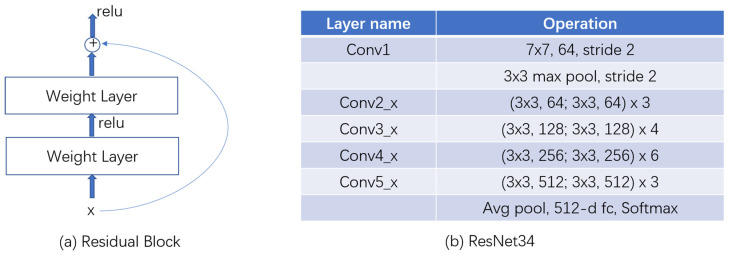
Residual block design and ResNet34 structure. (**a**) Residual block; (**b**) ResNet34.

To address the degradation problem, He et al. [13] created ResNet to learn residual representation between model input and output. ResNet consists of a series of residual blocks (Figure 3 left). Each block could be expressed as two components, identity mapping and residual part, and the latter is made up of two convolution layers.

Residual blocks bypass convolutional operation by skipping identity mapping, as the following Equation (4) shows:

(4) $$x_{l}=H_{l}\left(x_{l-1}\right)+x_{l-1}.$$

Based on residual blocks, we created ResNet34, which consists of 16 blocks (Figure 3). The input image is first resized as 672 × 672, and then it is convoluted by a 7 × 7 kernel into 64 channels with stride 2. A maximum pooling layer with a 3 × 3 kernel processes the acquired feature map with stride 2. The following is 16 residual blocks, three blocks with 64 output channels, four blocks with 128 output channels, six blocks with 256 output channels and three blocks with 512 output channels. Finally, after an average pooling, the feature map is flattened into a vector with 512 dimensions, and a successive fully connected layer functions as a 2-category classification head.

ResNet34 is supervised by Cross Entropy Loss [29]. Let *p*(*x*) be the ground truth probability distribution, *q*(*x*) a predicted probability distribution and *n* the number of categories. Cross Entropy Loss function between *p* and *q* could be represented as Equation (5):

(5) $$H\left(p,q\right)=-\Sigma_{i=1}^{n}p\left(x_{i}\right)\log\left(q\left(x_{i}\right)\right).$$

#### 2.2.2. DenseNet

**Figure 4:**
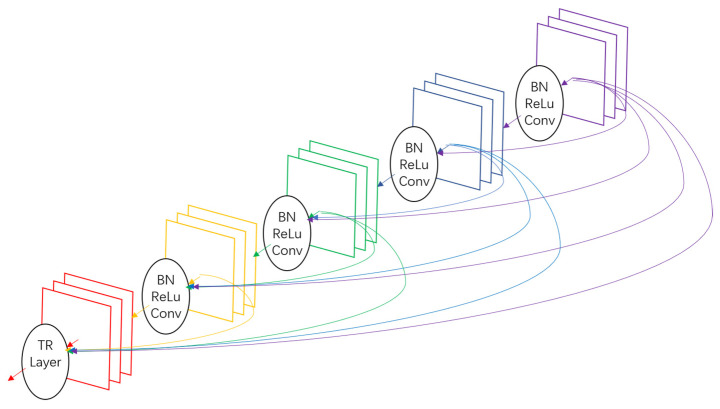
A dense block composed of 5 layers, each of which takes all precursor feature maps as input.

Similar to residual block, which creates short paths from precursor residual block to successive block, a dense block in DenseNet [14] connects any layer to all subsequent layers to address the degradation problem, as shown in Figure 4. These connections link layers densely, and thus gradients directly obtained from all preceding layers, $x_{0},x_{1},\ldots ,x_{l-1}$, enable deep supervision of $x_{l}$. Dense blocks concatenate feature maps from all precursor layers and then put them into a batch normalization (BN) [30], a rectified linear unit (ReLU) [26] and a 3 × 3 convolution layer (Conv), which could be represented as the following Equation (6):

(6) $$x_{l}=H_{l}\left(\left[x_{0},x_{1},\ldots ,x_{l-1}\right]\right).$$

**Figure 5:**
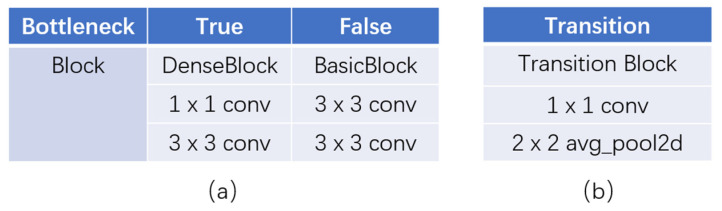
Details in DenseNet structure. (**a**) Bottleneck design; (**b**) transition block.

To lessen the number of episodic feature maps and to speed up calculations, a 1 × 1 convolution is introduced before 3 × 3 convolution, and such design is named bottleneck. Figure 5a makes a comparison between a dense block with bottleneck and a basic one.

In DenseNet, transition block plays a role in down-sampling feature maps instead of a single pooling layer. The transition blocks are composed of a batch normalization, a 1 × 1 convolution (Conv) and a 2 × 2 average pooling layer, as shown in Figure 5b.

**Figure 6:**
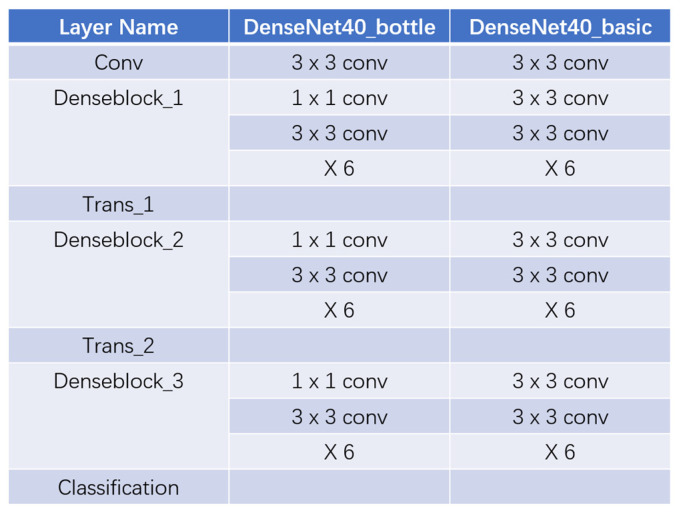
The whole structure of DenseNet40 with bottleneck dense block and with basic dense block, respectively.

Consisting of the components above, DenseNet40 with three bottleneck dense blocks or with three basic dense blocks is shown as Figure 6, left or right, respectively. DenseNet is also supervised by Cross Entropy Loss when completing a classifying task.

## 3. Results

### 3.1. Rebalanced Training for ResNet

In this research, we utilized four metrics to measure and compare the performance of our model’s accuracy, precision, recall and F1 score. Their definitions are shown in Table 1.

In the clinical practice of pneumoconiosis diagnosis, recall is often more important than precision, for the cost of a false negative is more than that of a false positive [31]. Therefore, we readjusted weights on each sample and assigned the weights of negative and positive samples to be different in the following experiment. Cross entropy loss function between the ground truth probability distribution p and the predicted probability distribution q could be represented as Equation (7), with a scale factor n balancing weights between negative and positive samples:

(7) $$Loss\left(y,\theta \right)=-\Sigma_{i=1}^{2}\left[\alpha p\left(x_{i}\right)log\left(q\left(x_{i}\right)\right)+\alpha \left(1-p\left(x_{i}\right)\right)log\left(1-q\left(x_{i}\right)\right)\right]\;\left(\alpha =n,\;if\;i=pos;\;\alpha =1,\;if\;i=neg\right).$$

In the following experiments, we set n=1 and n=5. While the former scale factor reflects the true distribution of samples in our dataset, the latter can intuitively rebalance the importance of negative and positive samples through sample weight.

**Figure 7:**
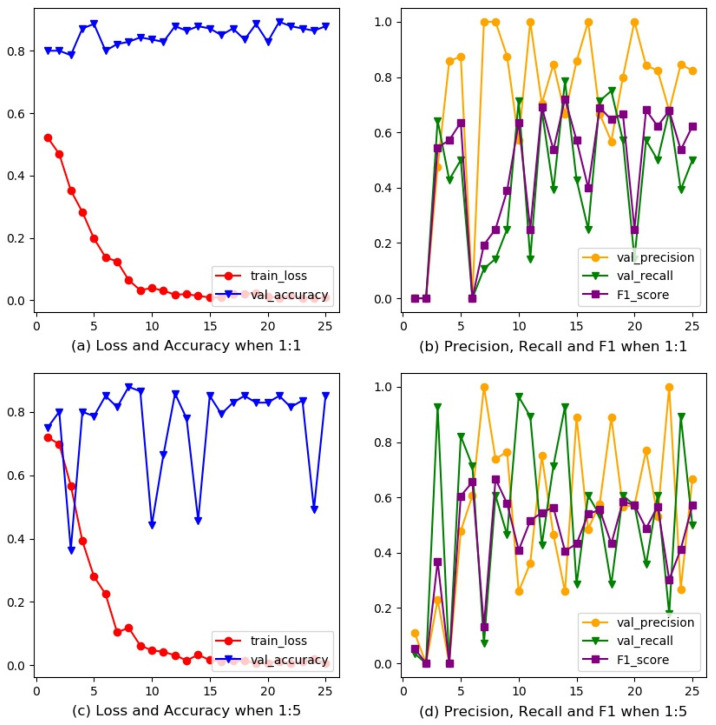
Training process of ResNet34. (**a**) Loss and accuracy when the scale factor is 1; (**b**) precision, recall and F1 score when the scale factor is 1; (**c**) loss and accuracy when the scale factor is 5; (**d**) precision, recall and F1 score when the scale factor is 5.

All training experiments were conducted based on PyTorch 1.2.0 after training 40 epochs with a batch size of 2, Adam optimizer and a learning rate of 0.00005. To improve the generalized performance on the test set, we only used random horizontal flip when training, rather than other data augmentation methods, for retaining basic characters of chest radiographs. Images were resized into 672 × 672 to fit the input size of our model. Figure 7a–d depicts the ResNet34 training process with scale factors 1 and 5, respectively. The final performance on the test set is shown in Table 2.

### 3.2. Refining Structure for DenseNet

In this section, we try to refine the model structure and improve the diagnostic accuracy of DenseNet from four perspectives, which are deep layers, dropout operation, reduction operation and bottleneck blocks. The scale factor of negative and positive samples is 5 in this section for rebalancing training. All training experiments were conducted based on PyTorch 1.2.0 after training 40 epochs with a batch size of 2, Adam optimizer and a learning rate of 0.00005. To improve the generalized performance on the test set, we only used random horizontal flip when training, rather than other data augmentation methods, for retaining basic characters of chest radiographs. Images were resized into 672 × 672 to fit the input size of our model.

#### 3.2.1. Deeper Layers

**Figure 8:**
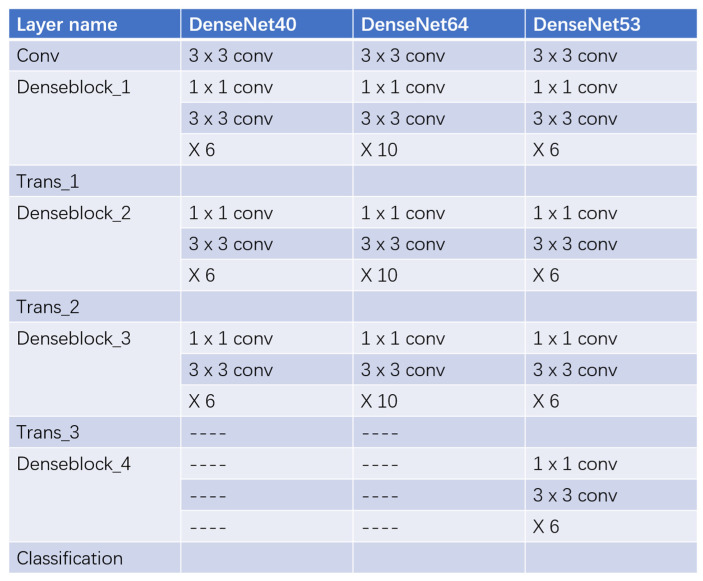
Structures of DenseNet40, DenseNet64 and DenseNet53. DenseNet64 has more layers within a dense block than DenseNet40, while the number of blocks is the same. DenseNet53 has more dense blocks than DenseNet40, though the number of layers within a block is the same.

More convolutional layers and deeper model structures could extract image features more effectively. Based on experiments, we improved the accuracy of pneumoconiosis detection by adding multiple layers within a dense block (DenseNet64) or tagging multiple dense blocks with the same inner structure (DenseNet53). The specific structures of DenseNet40, DenseNet64 and DenseNet53 are shown in Figure 8, and a brief comparison of their final performance on the test set is shown in Table 3. As DenseNet goes deeper, the accuracy of the pneumoconiosis diagnosis increases, no matter if the number of inner layers is elevated or if the number of dense blocks is promoted.

We selected the most promising DenseNet among the three, Densenet53, for further promotion in subsequent experiments.

#### 3.2.2. Dropout Operation

Dropout is a technique in which neurons are randomly inactivated with a certain probability to reduce model overfitting when training. We tested the impact of dropout on DenseNet53, shown in Table 4. The experimental results supported not to conduct dropout operation in subsequent experiments.

#### 3.2.3. Reduction Operation

Reduction operation works in transition layers to indicate how many times the number of output channels in next dense block is reduced. The impact of reduction is shown in Table 5, which supported a lower reduction rate to improve diagnostic accuracy.

#### 3.2.4. Bottleneck Blocks

As stated in Section 2.2.2, bottleneck blocks could lessen the number of input feature maps and thus speed up the calculation Verified by our experiment, the deep convolutional model with bottleneck blocks was 5% higher in detection accuracy than the model without bottlenecks, as shown in Table 6.

## 4. Discussion

In the above experiments, we trained and tested ResNet and DenseNet with different structures and parameters. Our training process adopted a rebalanced sample weight, which corresponds more with clinical practice. Under rebalanced training, ResNet34 achieved a 3.6% higher recall rate by only losing 1.4% in accuracy, and it finally reached 0.879 in accuracy when testing. This demonstrates that deep learning methods outperformed texture analysis [32], which achieved an accuracy of 0.793 on diagnosing 29 images.

For DenseNet, we added deep layers, changed bottleneck blocks, conducted a dropout operation and examined a reduction operation to enhance feature representation ability. As results show above, deeper layers and a lower reduction rate significantly improved the diagnostic accuracy by 4.3% and 5%, respectively. Both improvements increased the number of model parameters and, thus, extracted more image information about pneumoconiosis. Bottleneck block also increased the detection accuracy by 5%, for a 1×1 convolution introduced in the bottleneck fused features despite cutting parameters down. Shown by experiments in Section 3.2.2, the dropout operation decreased accuracy. The reason is that in 40 epochs, which is restricted for comparison, DenseNet53 with dropout cannot be trained finely, and it also demonstrates a drawback for consuming too much time when training under limited calculation resources. Finally, the best was DenseNet53 with bottleneck blocks, which had a 0.25 reduction rate and no dropout operation, reaching 0.886 in accuracy when testing.

**Figure 9:**
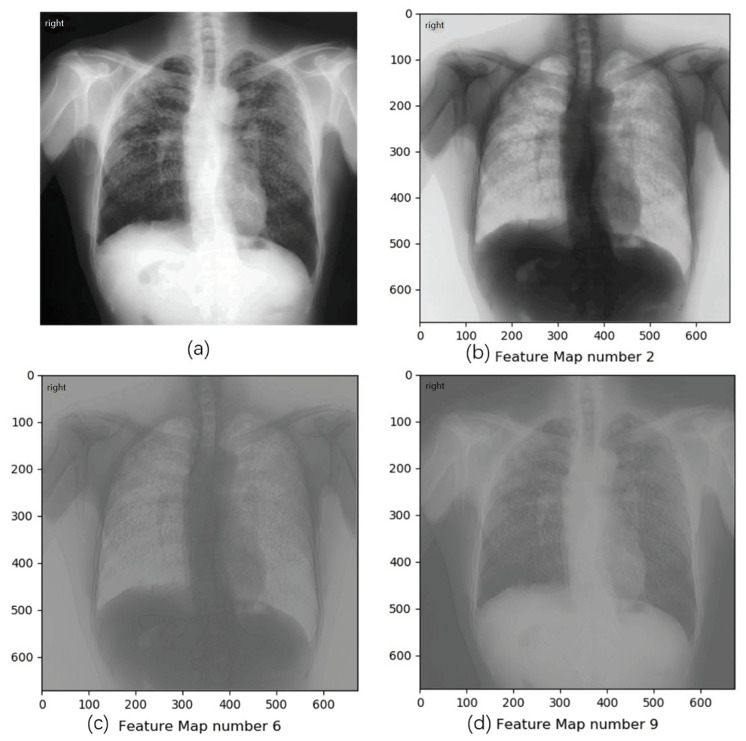
A pneumoconiosis-positive sample in the dataset and visualizations of feature maps from several channels in DenseNet53. (**a**) The original radiograph; (**b**) the 2nd feature map, which separates lung region to a certain extent; (**c**,**d**) 6th and 9th feature map, which capture and highlight suspected opacities in pneumoconiosis radiograph.

To study whether CNNs really learned features, we visualized and analyzed the feature map output by the first convolutional layer of trained DenseNet53. The feature map refers to each channel of the output tensor from convolutional layers. After training, a feature map can be regarded as the detection of one radiograph feature related to pneumoconiosis. The strength of a pixel value in the feature map is the response to the strength of feature. By visualizing these feature maps, our system could show signs of pneumoconiosis and, thus, provide a reference to radiologists. Results demonstrate that several output channels in DenseNet53, for example, the 2nd feature map in our trained model, can partially separate lung region from radiographs, shown in Figure 9b. Other channels could capture and highlight suspected opacities, which are typical features of pneumoconiosis, shown in Figure 9c,d. These visualizations of feature maps could enhance the interpretability of deep learning models in medical diagnosis.

This study focuses on pneumoconiosis diagnosis, specifically whether a patient has pneumoconiosis or not. In future research, we will apply high-accuracy and interpretable diagnosis techniques based on CNNs into pneumoconiosis staging, which concentrates on determining each subject (normal, stage I, II or III pneumoconiosis) [32]. Staging is more challenging because of small differences between stages, and it is also more practical when accurately determining medical insurance.

## 5. Conclusions

Pneumoconiosis is one of the most common occupational diseases in China, with a high incidence and high treatment cost, which has caused huge economic losses to modern healthcare systems and patient families. Currently, the diagnosis of pneumoconiosis is still largely dependent on the experience of radiologists, which affects the early diagnosis among huge populations. Recent research has focused on computer-aided detection based on machine learning algorithms, among which artificial neural network (ANN) has achieved high accuracy. However, due to the imbalance of samples and lack of interpretability, deep learning models are difficult to be widely used in clinical practice.

To solve these two problems, we first aggregated a pneumoconiosis radiograph dataset including both negative and positive samples. Second, we implemented, improved and compared ResNet and DenseNet, two typical deep convolutional approaches in pneumoconiosis detection, and we adopted balanced training using re-adjusted weights. Comparative experiments and the ablation study conducted on the above dataset demonstrated high accuracy (88.6%). Third, we visualized feature maps to show suspected opacities on pneumoconiosis radiographs, which could enhance interpretability of deep learning models and provide solid diagnostic reference for surgeons.

## Figures and Tables

**Table 1:** Definitions of metrics utilized.

Metrics	Definition
TP	True Positive. Samples predicted to be positive with a positive ground truth label.
FP	False Positive. Samples predicted to be positive with a negative ground truth label.
FN	False Negative. Samples predicted to be negative with a positive ground truth label.
TN	True Negative. Samples predicted to be negative with a negative ground truth label.
Accuracy	$\mathrm{accuracy}=\frac{\mathrm{TP}+\mathrm{TN}}{\mathrm{TP}+\mathrm{TN}+\mathrm{FP}+\mathrm{FN}}$
Precision	$\mathrm{precision}=\frac{\mathrm{TP}}{\mathrm{TP}+\mathrm{FP}}$
Recall	$\mathrm{recall}=\frac{\mathrm{TP}}{\mathrm{TP}+\mathrm{FN}}$
F1 Score	$F1=\frac{2*\mathrm{precision}*\mathrm{recall}}{\mathrm{recall}+\mathrm{precision}}$

**Table 2:** Performance of ResNet34 on test set with scale factors 1 and 5, respectively.

	ResNet34	ResNet34
n	1	5
Accuracy	0.893	0.879
Precision	0.842	0.739
Recall	0.571	0.607
F1 Score	0.681	0.667

**Table 3:** Performance of DenseNet40, DenseNet64 and DenseNet53 on the test set.

Model	DenseNet40	DenseNet64	DenseNet53
Accuracy	0.843	0.871	0.886
Precision	0.714	0.750	0.833
Recall	0.357	0.536	0.536
F1 Score	0.476	0.625	0.652

**Table 4:** Impact of dropout operation on DenseNet53.

Drop rate	0	0.25
Accuracy	0.886	0.843
Precision	0.833	0.714
Recall	0.536	0.357
F1 Score	0.652	0.476

**Table 5:** Impact of reduction operation on DenseNet53.

Reduction	0.25	0.5	1
Accuracy	0.886	0.871	0.836
Precision	0.833	0.692	0.647
Recall	0.536	0.643	0.393
F1 Score	0.652	0.667	0.489

**Table 6:** Impact of bottleneck blocks on DenseNet53.

Bottleneck	False	True
Accuracy	0.836	0.886
Precision	0.600	0.833
Recall	0.536	0.536
F1 Score	0.566	0.652

## Data Availability

The data presented in this study are available on request from the corresponding author.
